# Fatigue Analysis of Welded Joints Using a Thin-Walled Al/Fe Explosive Welded Transition Joints

**DOI:** 10.3390/ma16186259

**Published:** 2023-09-18

**Authors:** Dominika Płaczek, Paweł Maćkowiak, Dariusz Boroński

**Affiliations:** Faculty of Mechanical Engineering, Bydgoszcz University of Science and Technology, 85-796 Bydgoszcz, Poland; pawel.mackowiak@pbs.edu.pl (P.M.); dariusz.boronski@pbs.edu.pl (D.B.)

**Keywords:** fatigue of materials, cruciform welded joint, transition joints

## Abstract

The study presents an analysis of S355J2+N steel and AA5083 aluminum alloy welded structural joints using explosion welded transition joints of reduced thickness. The transition joint thickness reduction significantly hinders the welding of the joints due to the risk of damage to the Al/steel interface as a result of the high temperatures during welding. In the previous article, the strength of the transition joint was analyzed but ship structures, apart from static loads, are subjected to many different cyclical loads. Welded structural joints are analyzed to determine the welding influence on the fatigue life and fracture type of the transition joints. The results of the fatigue tests show that the fatigue damage in the specimens occurs in the aluminum welded joint, and not in the explosively welded joint. The damage obtained was characteristic of cruciform welded joint specimens and both types of root and toe damage occurred. Based on the obtained results, fatigue curves for the joint were determined and compared to the fatigue curves for the AA5083 base material. The experimental fatigue curve was also compared with the design curve for welded aluminum structures from Eurocode. The conducted analysis showed the possibility of using Al/steel explosion welded transition joints of reduced thickness to transfer cyclical loads.

## 1. Introduction

Optimization of structure in terms of weight reduction and production costs is carried out by combining dissimilar materials into a multi-material hybrid structure. Joining materials with different mechanical and metallurgical properties requires systematic approach to material selection: these materials will interact with each other in new ways, and new manufacturing systems might be needed [1]. The combination of steel with aluminum alloys is known in literature and industry. There are many different methods of joining dissimilar materials. For example, methods such as adhesive bonding or friction welding can be mentioned. In the case of adhesive bonding, the problem is the strength and durability of the joint, especially in the case of a small joining surface [2]. When joining materials using friction welding, it is difficult to obtain large-surface joints in the form of sandwich sheets [3]. A much more efficient method of obtaining sandwich sheets is explosion welding. A combination of various materials can also be obtained by welding joints using explosive welding transition joints (TJ) [4]. This method connects large-sized elements using welding, which is a technique common in industry. An example of such a solution is joining a steel hull with an aluminum superstructure using bimetallic strips [5,6]. Other exemplary applications in the shipbuilding industry are presented in the papers [7,8,9]. 

Insufficient fatigue strength of welded joints is one of the most common causes of damage. Considering that welding is a source of notches resulting from geometric and material discontinuities, the fatigue strength of the welded joints is lower than that of the base material (BM) [10]. The result is fatigue crack initiation points due to high stress or strain concentration in the notch zones. This applies to most types of welded joints, including the load-carrying cruciform welded joints (LCWJ), one of the most common types of welded joints used in shipbuilding. There are many global and local approaches to assessing the fatigue life of welded structures, including: notch stress [11], hot spot stress [12], equivalent structural stress method [13], notch stress intensity factor (NSIF) method [14,15], strain energy density (SED) method [10,16], peak stress method (PSM) [17,18], and fracture mechanics method [19]. Henk den Besten presented a classification of fatigue damage criteria, modeling, development, and trends in welded joints from the area of marine structures [20]. The author points out that fatigue is usually the valid limit state for marine structures and classifies fatigue failure criteria developed over time in relation to the different weld and environmental parameters.

The current state of knowledge regarding approaches for predicting the fatigue life of welded joints used for the marine industry and the latest advances in welding dissimilar materials was demonstrated by Corigliano et al. [21]. The authors state, among others, that the need for using different materials to optimize weight and structural performance of ships and marine structures is rapidly increasing and the most used type of dissimilar welded joints is nowadays represented by the Al/Steel type obtained through the use of explosion welding (EXW). Moreover, authors indicate that the current codes on the fatigue design of welded structures, which are accepted by some of the major Ship Classification Societies, are based on the nominal stress approach. The current codes report the S–N curves of the different fatigue class of the welded joints taking into account geometry of the joint and loading mode. However, Classification Societies and Ship Registers define categories of fatigue strength only for homogeneous welded joints. This is also confirmed by the work of Meneghetti et al. [22]. Therefore, it is not possible to relate the results of fatigue life tests of dissimilar joints with the design curves for fatigue resistance classes (FAT) presented by the International Institute of Welding in Recommendations for Fatigue Design of Welded Joints and Components [23].

The paper presents a fatigue analysis of thin-walled welded joints of steel and aluminum alloy with the use of explosively welded transition joints of limited thickness. Strength analysis of S355J2+N steel and AA5083 aluminum alloy welded structural joints using explosion welded transition joints of reduced thickness was demonstrated by Boroński et al. [24]. This work is a continuation of the study of welded joints of aluminum alloy steels using a thin-walled explosively welded transition joint in the aspect of fatigue life analysis using S–N approach and is the next phase of a wider research program for this type of structures.

## 2. Materials and Methods

### 2.1. Material and Specimens Preparation

The materials used for the tests were aluminum alloy AA5083 in temper H321 and 355J2+N steel. In the explosive welding process, the intermediate layer between these materials was aluminum AA1050 in the H24 temper. The chemical composition of individual materials obtained from the manufacturer is presented in Table 1. The mechanical properties of the materials accepted for testing are presented in Table 2. 

AA5083 alloy subjected to strength marine tempers H321 is an alloy with a content of about 5% magnesium, characterized by low sensitivity to cracking and very good welding properties [25]. The material is characterized by high resistance to intercrystalline corrosion and sea water. In the production of vehicles, its main application is tankers; bodies and structural elements used in interior development. It is also one of the basic construction materials in the shipbuilding industry, used, among others, in the hulls and superstructures of ships [26,27,28]. 

S355J2+N steel is an unalloyed, low-carbon, high-strength structural steel. Currently, steels are the most commonly used group of engineering materials and are used in various industries, i.e., construction of bridges, buildings, ships, cars, rail vehicles. Due to good mechanical properties, easy processing, forming, good weldability, and crack resistance as well as low price, structural steels are widely used in industry. S355J2+N steel is most often used for the production of load-bearing parts of structures exposed to dynamic loads and low temperatures [29,30].

AA1050 in the H24 temper was used as an intermediate layer between the AA5083 alloy and the S355J2+N steel to join them in the explosive welding process. This material is characterized by high plasticity and corrosion resistance [31]. 

**Figure 1:**
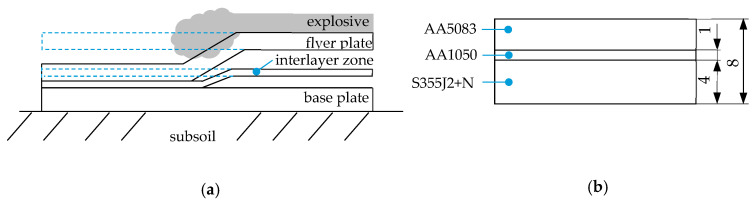
Explosive welding: (**a**) process; (**b**) dimensions of the explosively welded plate.

The possibility of joining steel with aluminum alloy by welding was realized with the use of Al/Fe explosively welded transition joints. The plates were welded in a parallel arrangement in which the flyer plate was at a constant distance from the base plate [32,33]. Testing plates were produced using the explosive material at a detonation velocity in the range of 1950–2050 m/s. The thicknesses of individual layers were respectively: 3 mm for AA5083, 1 mm for AA1050, and 4 mm for S355J2+N. The explosive welding process and the dimensions of the explosively welded plate are shown in Figure 1.

Sheet formats for welding were cut by abrasive blasting. This technology generates a very small amount of heat, so it did not affect the strength and structure of the materials. The width of the explosively welded transition joint is four times the thickness of a single sheet. This dimension results from the increase in the surface area of the AA1050 transition joint, which has more than two times lower strength than AA5083. In addition, this width allows you to maintain an appropriate distance from the edge during the sheet metal welding process and at the same time provides the possibility of greater heat dissipation [24]. 

**Figure 2:**
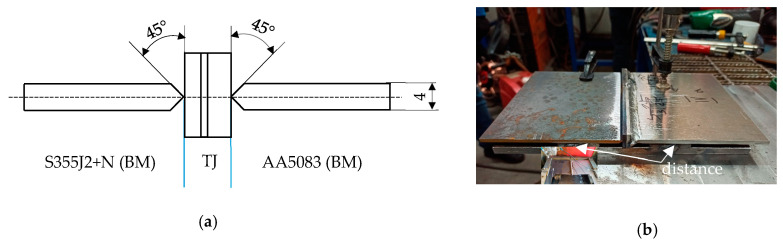
Welding process: (**a**) preparation of sheets; (**b**) welding on the jig.

Welding was carried out using the GMAW method using the TPS400i FRONIUS device (Fronius, Wels, Austria). According to the tests carried out, aluminum alloy sheets were first welded to the explosively welded transition joint [24]. The aluminum alloy was welded using method 131, Metal Inert Gas (MIG). The welds were made with AlMg5 welding wire with a diameter 1.2 mm (EN ISO 18273: S Al 5356, AWS A5.10: ER 5356 [34]) in a gas shield Ar 2.2 (22 L/min). The adopted sequence of welding causes the possibility of greater heat dissipation resulting from the increased surface area of the structure. Limitation of heat introduced in interface zone aims to minimize the growth of brittle intermetallic phases and maintain mechanical properties explosive welded transition joint [35]. Before welding, the materials were mechanically cleaned. The aluminum alloy was degreased with a remover. The steel was welded using method 135, Metal Active Gas (MAG). For welding the steel, a Multimet IMT3 wire with a diameter of 0.8 mm was used (EN ISO 14341-A-G 4Si1, AWS A5.18-ER70S-6 [36]) in a M21 gas shield (82% Ar + 18% CO_2_, 25 L/min). Figure 2a shows the preparation of sheets for welding. The welding process was carried out in a device that ensured axial welding of the sheets to the explosively welded transition joint (Figure 2b). Welding parameters are included in Table 3.

**Figure 3:**
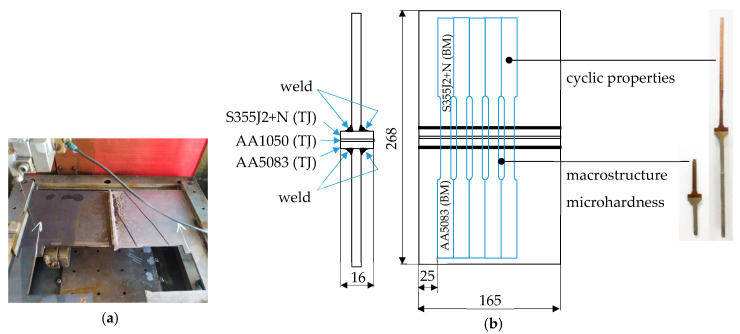
Specimens preparation: (**a**) specimens cutting method WEDM; (**b**) specimens along with their purpose.

Specimens were cut out by the wire electrical discharge machining method (Figure 3a). The diagram of the welded sheets, the place of cutting the specimens, and their purpose are shown in Figure 3b.

### 2.2. Methods

To reveal the zones resulting from the welding process, a macrostructural analysis of metallographic specimens was carried out using an optical microscope. The Keller followed by Weck solutes was selected to etch the aluminum alloy side, a steel 5% solution HNO_3_ C_2_H_5_OH. The process was performed at room temperature to reveal the macrostructure of the welded specimens and then washed with water and acetone, and then air-dried.

The Shimadzu HMV-G20DT hardness tester (Shimadzu, Kioto, Japan) was used to measure the microhardness. The measurement was made on the cross-sectional area of the explosively welded transition joint before and after the welding process.

**Figure 4:**
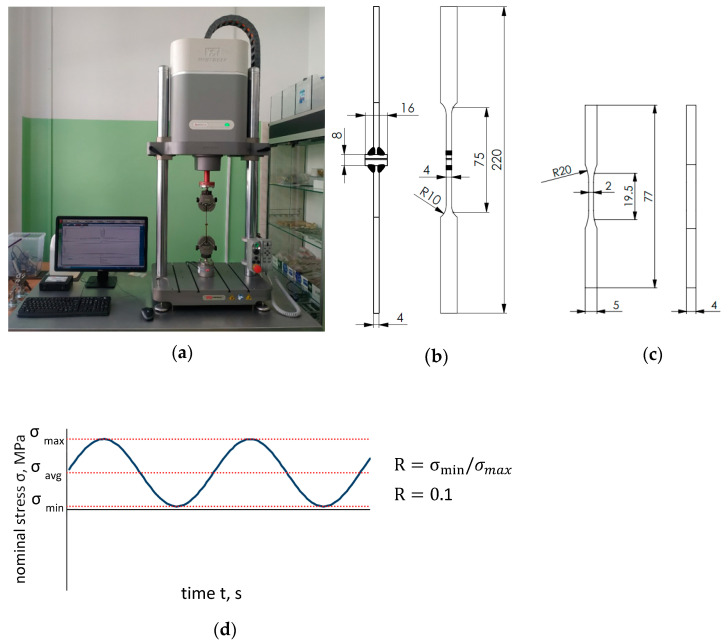
Testing: (**a**) Instron ElectroPuls E3000 test stand; (**b**) specimen of Al/steel joint; (**c**) specimen of BM AA5083; (**d**) diagram of a sinusoidal variable load R=0.1.

Fatigue tests of Al/steel welded specimens and AA5083 alloy BM were carried out on an Instron ElectroPuls E3000 testing machine (Instron, Norwood, MA, USA) (Figure 4a). The dimensions of the specimens are shown in Figure 4b,c. The specimens were loaded with a sinusoidally variable load, so as to exclude the possibility of a compressive force due to the possibility of its buckling. The cycle asymmetry coefficient as the ratio of the minimum stresses in the cycle to the maximum stresses in the cycle was R = 0.1. 

The analysis of fatigue fractures in the BM AA5083 and Al/steel welded joints was performed on the JEOL 6480LV device (JOEL, Tokio, Japan). The specimens were cleaned with alcohol and dried with compressed air. The specimens fixed in the holder were placed in the chamber of the scanning microscope.

## 3. Results and Analysis

### 3.1. Macrostructure of Welded Joints

**Figure 5:**
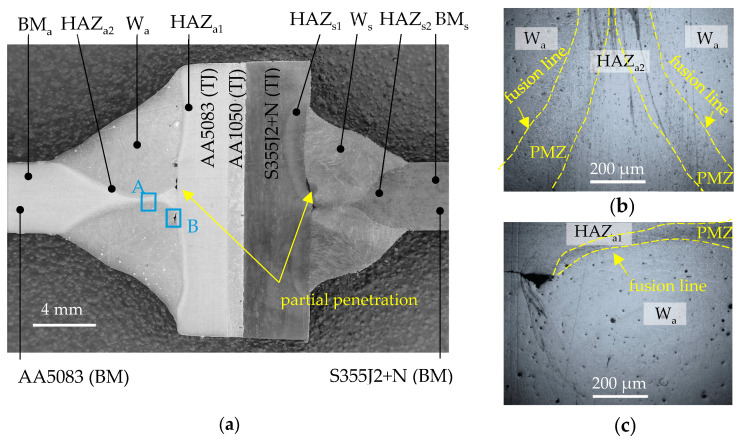
(**a**) Al/steel joint macrostructure with post-weld zone designation; (**b**) area A; (**c**) and B.

Figure 4 shows an example image of the joint macrostructure with the heat affected zones (HAZ), the partial fusion zone (PMZ), and the weld (W) marked. Base materials (BM) and explosively welded transition joint (TJ) are also marked. Partial penetration is visible in the welds on the side of aluminum alloy and steel (Figure 4a). In the PMZ, the individual grain melted partially, which might lead to liquation cracks. The size of the HAZ from both the BM and TJ sides in AA5083 does not exceed 2 mm. In the macrostructural analysis, the influence of welding on the transition zone between the AA5083 and S355J2+N layers in TJ is not observed. Slight grain boundary flow was observed in PMZ on the side of AA5083 alloy (Figure 5b,c). 

### 3.2. Microhardness Distributions

**Figure 6:**
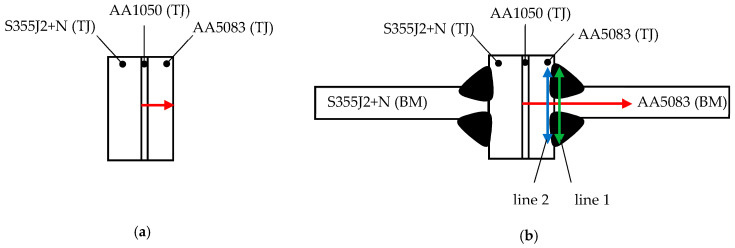
Measurement points of hardness distribution: (**a**) before welding; (**b**) after welding.

**Figure 7:**
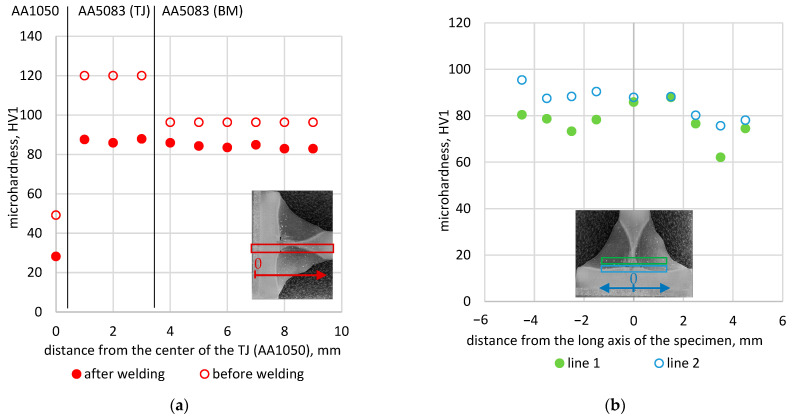
Microhardness distribution: (**a**) along the longitudinal axis of the specimen; (**b**) along the transverse axis of the specimen.

Measurement points of hardness distribution shown in Figure 6. The graphs (Figure 7) show the results of the hardness test before and after the welding process.

The results of the microhardness measurement, as expected, showed a decrease in hardness after the welding process. Average values from measurements in individual zones are presented in Table 4. In the explosively welded fitting, the hardness decreased by about 42%; for AA5083 TJ, by 27%. For the AA5083 BM area, the difference was the smallest and amounted to 14%.

Comparing the microhardness distributions along the transverse axis of the specimen, one side of the specimen has a greater hardness than the other (Figure 7b). This indicates the order in which the welds are applied. An increase in temperature leads to grain spreading, which in turn leads to a decrease in microhardness. The side on which the weld was made first has a lower microhardness.

### 3.3. Fatigue Analysis in Terms of Stress

**Figure 8:**
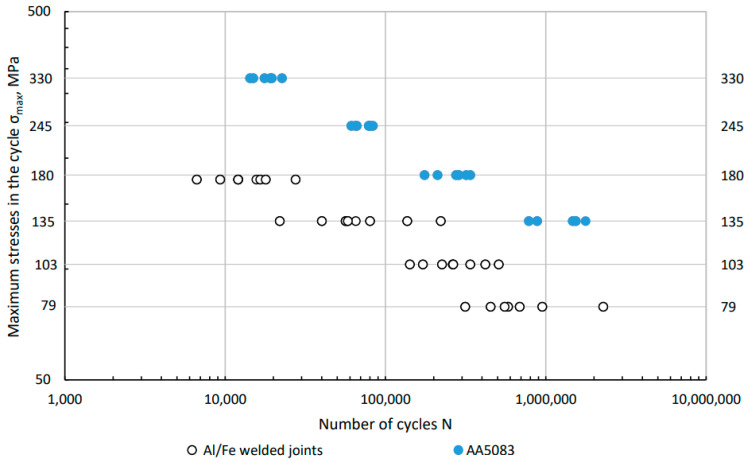
Fatigue life of the Al/steel welded joint in relation to the fatigue life of the BM AA5083.

Based on the preliminary test results, four load levels were established for Al/Fe joint specimens and AA50083 BM. In all, 32 specimens of the Al/steel joints and 26 specimens of the BM of the AA5083 alloy were tested. The results of individual tests, together with the determination of the fatigue failure point, are presented in Table 5 and in the Figure 8, respectively.

Fatigue life diagrams are described by the equation:

(1) $$log(N)=m\cdot \log\sigma_{max}+A$$

where:

*m*—slope of the linear regression
*A*—constant of the linear regression

**Figure 9:**
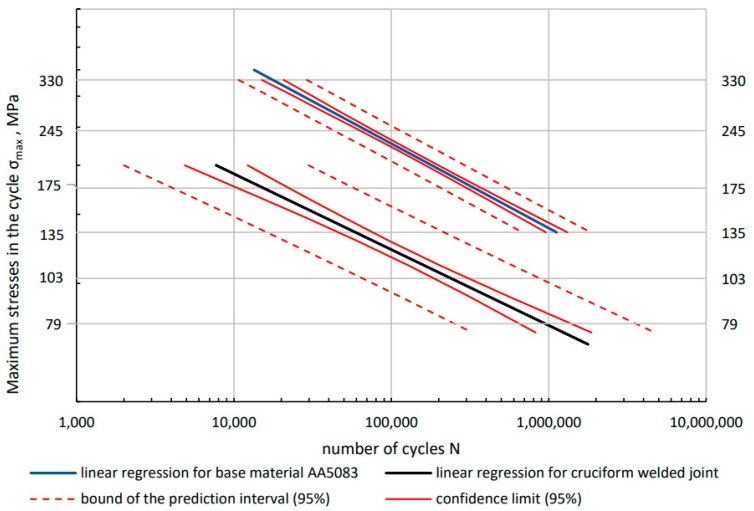
Fatigue diagrams for the BM AA5083 and the transition joint Al/steel determined on the basis of experimental tests with the statistical method of confidence interval for linear regression and prediction interval for *p* = 0.95.

The *m* and *A* values determined by regression analysis of the test results are shown in Table 6, and their courses for the aluminum alloy and joint are shown in Figure 9. Based on the designated confidence intervals for both linear regressions, it can be assumed that they are parallel to each other. Assuming the conventional fatigue limit at N = 5 × 10^6^ [37], [38] a stress value of 99.5 MPa is obtained for the BM, and 63.1 MPa for the Al/steel joint. The conventional fatigue limit for the Al/steel combination is therefore 36.4% lower than for the AA5083 BM. The dashed red lines indicate the prediction area for *p* = 0.95. Confidence intervals (*p* = 0.95) for the determined regressions are marked with solid red lines. The coefficient of determination R^2^ for the BM AA5083 is 0.98, which proves that the determined regression is well matched to the obtained results. For Al/steel joints, the coefficient of determination R^2^ is 0.87. These specimens are characterized by a much larger scatter of results, which is also indicated by a wider prediction range.

**Figure 10:**
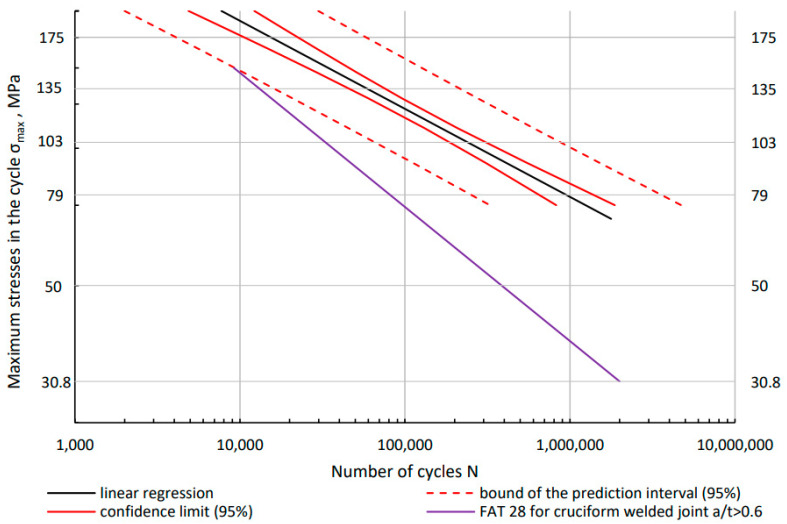
Comparison of the fatigue diagram determined experimentally for the Al/steel transition joint with the S–N design diagram made on the basis of EN 1999-1-3: Eurocode 9 [39].

The obtained test results were compared to the design diagram (Figure 10). For aluminium alloys, corresponding S–N curves were applied with reduced reference values. The S–N curves represent the lower limit of the scatter band of 95% of all test result available considering further detrimental effects in large structures. Referring to EN 1999-1-3: Eurocode 9 [39] and IIW standard, the design curve for aluminum was determined. The standard gives two values that allow you to determine the design curve. $\Delta \sigma_{c}$ is reference fatigue strength at 2 × 10^6^ cycles (normal stress) being between the maximum and minimum stress in the cycle and m1 is inverse slope of logΔ*σ*-log*N* fatigue strength curve value. For the cruciform welded joint in EN 1999-1-3: Eurocode 9, these values are respectively $\Delta \sigma_{c}$ = 28 MPa and m_1_ = 3.4. Based on these two values, the design curve can be derived.

The S–N curve represent section-wise linear relationships between log (Δσ) and log(N).

log(*N*) = *A* + *m* · log(Δ*σ*) (2)

where:

*m*—slope exponent of S–N curve
*A*—coefficient
Δ*σ*—nominal stress range (normal stress)
*N*—total number of stress range cycles

By rearranging the equation, the value of the intercept A can be determined for the known value of *N_c_*, Δ*σ_c_*, and *m*.

(3) $$A=\log N_{c}\;-m\cdot \log∆\sigma_{c}$$

where:

*N_c_*—number of cycles (2 × 10^6^) at which the reference fatigue strength is defined
$\sigma_{c}$—reference fatigue strength

For the data $\Delta \sigma_{c}$ = 28 MPa, for the cycle asymmetry coefficient R = 0.1, ${\Delta \sigma }_{cmax}=30.8$ MPa. With the number of cycles N = 2 × 10^6^ and m_1_ = 3.4, the value of A is 11.36. Based on these values, the design curve (Figure 10) was determined. It is below the lower prediction limit of the experimentally determined points. For higher load levels and low number of cycles (below 10,000 cycles), it is better to use deformation calculation methods.

**Figure 11:**
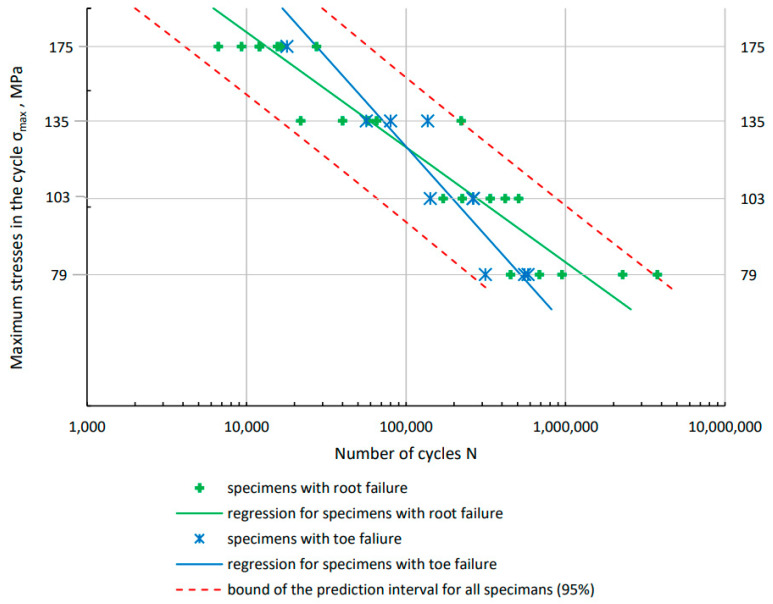
Fatigue test results with separate marking of specimens and determined regression lines for two types of damage to specimens: root and toe.

The graph (Figure 11) shows the results of fatigue tests depending on the mechanism of specimen failure: root type and toe type. Regression lines were determined separately for both types of failure. These lines intersect at the number of cycles N = 10^5^. Greater durability for higher load levels is obtained with toe-damaged specimens. At lower stress values, specimens characterized by root failure are more durable.

### 3.4. Analysis of Fatigue Fractures

Images of fatigue fractures of the specimens were made for specimens of Al/steel welded joints. The specimen for which the photos were taken were damaged at the toe. An additional Energy Dispersive Spectroscopy analysis was performed for the Al/steel welded joint. The reference for the analysis of welded joints are the photos of fatigue fractures of specimens from the BM AA5083.The possibility for observing in the secondary (SEI) and backscattered electrons modes (BEC) is demonstrated.

**Figure 12:**
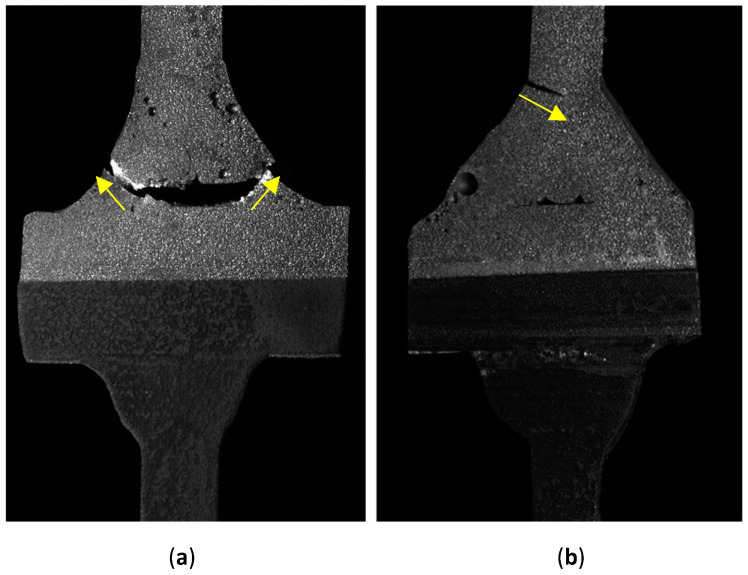
Fatigue failure points at: (**a**) root; (**b**) toe.

As shown in Table 4, the nature of the destruction of the specimens was twofold. As a result of fatigue tests, the specimens were damaged at the root or toe point. The typical weld toe and weld root failure modes obtained after fatigue tests are shown in Figure 12. The percentage ratio of ‘at toe damage’ to ‘all tested specimens’ of Al/steel welded joints is 31%.

**Figure 13:**
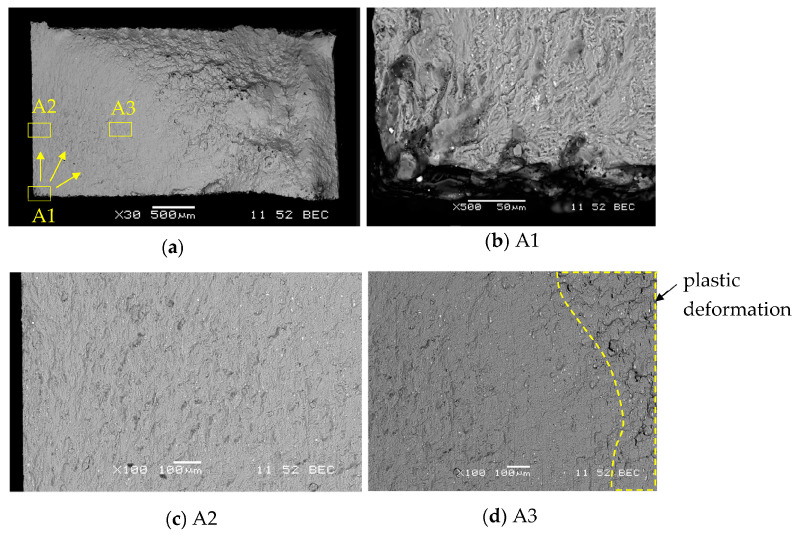
SEM images of the fracture surface of fatigue test specimens of AA5083 (specimen AA5083_3): (**a**) photo of the specimen with indication of the crack propagation direction; (**b**) fatigue crack initiation point; (**c**) area of narrow fatigue lines; (**d**) wide lines with the area of plastic deformation.

The fatigue fracture of the AA5083 BM is shown in Figure 13a. The places subjected to a more thorough analysis (A1, A2, A3) were also marked. The arrows indicate the directions of crack propagation. By analyzing the fatigue fractures of the BM AA5083, the place of crack initiation was indicated (Figure 13a). The initiation occurred in one of the corners of the specimen (Figure 13b). The direction of crack propagation from one point is visible. Fatigue striations are visible in the A2 and A3 areas. The striations become wider as the fatigue crack progresses (Figure 13c,d). The zone of plastic deformation is also visible in the A2 area.

**Figure 14:**
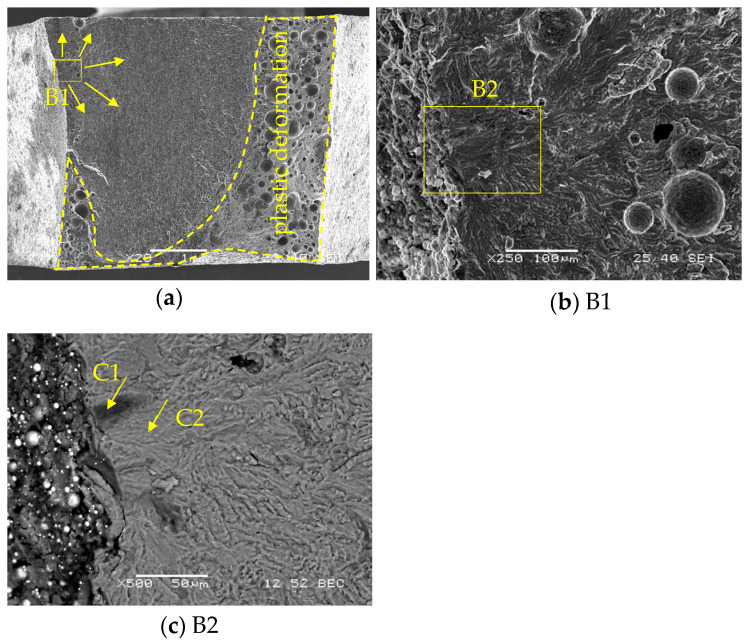
SEM images of the fracture surface of fatigue test specimens of Al/steel (specimen Al/steel_8): (**a**) welded joints with fatigue failure points at the toe; (**b**) fatigue crack initiation site; (**c**) marking points for EDS analysis.

**Figure 15:**
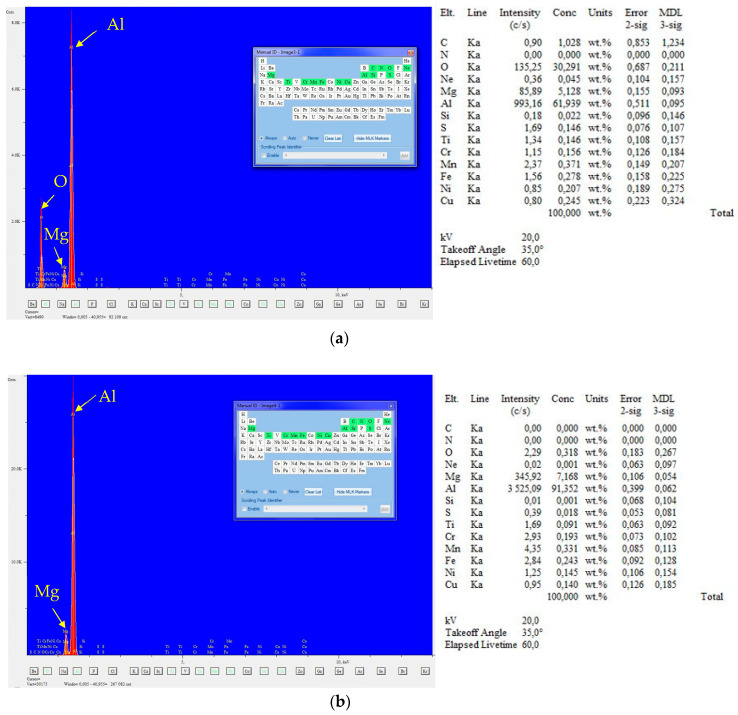
EDS spot analysis of the points marked in Figure 13c: (**a**) C1; (**b**) C2.

Figure 14a shows SEM images of the fracture surface of fatigue test specimens of Al/steel welding joints with fatigue failure points at the toe. The place of initiation of the fatigue crack B1 and the direction of crack propagation are marked. The area of plastic deformation was marked. The fatigue crack initiation site is shown in Figure 14b. In Figure 14c, a darker area was observed using the BEC detector and verified for the crack initiation site. This site was analyzed for chemical composition (area C1). For comparison, an analysis was performed for the bright C2 area. In both areas, there is a clear presence of two basic elements for the AA5083 aluminum alloy: Al and Mg (Figure 15a,b). EDS analysis at site C1 additionally shows the presence of oxygen in this area.

## 4. Conclusions

The paper presents the results of the next phase of testing thin-walled welded joints of steel and aluminum alloy with the use of TJ regarding their fatigue analysis in terms of S–N. On the basis of the conducted research, several conclusions and observations were formulated.

- Minimized thickness of the explosive welded transition joints makes welding much more difficult due to the risk of damage to the Al/steel interface as a result of the high temperatures during welding. None of the Al/Fe welded joints subjected to fatigue tests failed in the AA1050 layer of the explosively welded transition joint. It can therefore be concluded that Al/steel welded joints can be successfully used with the use of explosively welded transition joint with reduced thicknesses.
- The obtained results of fatigue tests of Al/steel welded specimens were compared with the results of the BM AA5083. The welded joint reduces the fatigue life compared to the performance of the BM. However, a comparison of the experimental results to the design curve showed that the results of the Al/Fe combination met the design requirements.
- As expected, the welding process caused a change in the hardness of the materials in the HAZ. The reason for this is grain growth caused by the introduction of heat during welding [24].
- Failure fractures were analyzed for specimens with the lowest load level. Slower fatigue failure shows the place of crack initiation and its propagation more clearly for BM AA5083 and Al/steel welded joint.

The next stage of the research will be the application of a local approach, taking into account local fatigue properties for individual zones of the analyzed joints.

## Figures and Tables

**Table 1:** Percentage of chemical composition of material in an explosively welded transition joint.

	C	Si	Mn	P	S	N	Al	Cu	Cr	Ni	Mo	Nb	Ti	V	Fe	Mg	Zn
AA5083	-	0.11	0.77	-	-	-	balance	0.09	0.06	-	-	-	0.01	-	0.31	4.7	0.01
AA1050	-	0.12	0.02	-	-	-	99.52	-	-	-	-	-	0.03	-	0.27	-	-
S355J2+N	0.14	0.05	1.5	0.015	0.001	0.006	0.038	0.03	0.04	0.04	0.01	0.01	0.002	0.004	-	-	-

**Table 2:** Mechanical properties of materials used for testing [24].

Material		$\sigma_{y}$, MPa	$\sigma_{u}$, MPa	$A_{5}$, %	E, MPa
Explosive welding transition joint (TJ)	S355J2+N	527	606	25	210,000
	AA1050	101	107	5	73,000
	AA5083	257	356	14	71,000
Base material (BM)	S355J2+N	370	524	27	207,300
	AA5083	225	362	15	77,000

**Table 3:** Welding parameters.

Weld	Welding Speed [mm/min]	I [A]	U [V]	Wire Feed [m/min]	Power P [W]
AA5083	541	136	18.6	7.4	2630
S355J2+N	436	145	19.5	10.4	2815

**Table 4:** Average values of microhardness measurements for individual areas before and after the welding process.

	HAZ	Weld	AA1050	AA5083 TJ	AA5083 BM
After welding	86.6	77.6	28.2	87.1	82.9
Before welding	-	-	49.2	120.0	96.3

**Table 5:** Fatigue life of specimens with fatigue failure point.

Al/Fe Welded Joints						AA5083 (BM)				
Number of Specimen	Stress σ	Number of Cycles N	log σ	log N	Fatigue Failure Points	Number of Specimen	Stress σ	Number of Cycles N	log σ	log N
	MPa	-	-	-			MPa	-	-	-
Al/steel_1	79	3,765,329	1.8976	6.5758	root	AA5083_1	135	1,536,149.00	2.1303	6.1864
Al/steel_2	79	947,946	1.8976	5.9768	root	AA5083_2	135	881,809.75	2.1303	5.9454
Al/steel_3	79	452,000	1.8976	5.6551	root	AA5083_3	135	782,027	2.1303	5.8932
Al/steel_4	79	686,373	1.8976	5.8366	root	AA5083_4	135	1,471,946.75	2.1303	6.1679
Al/steel_5	79	2,278,247	1.8976	6.3576	root	AA5083_5	135	1,768,942	2.1303	6.2477
Al/steel_6	79	581,135	1.8976	5.7643	toe	AA5083_6	135	1,009,250	2.1303	6.004
Al/steel_7	79	552,342	1.8976	5.7422	toe	AA5083_7	180	211,199.75	2.2553	5.3247
Al/steel_8	79	313,957	1.8976	5.4969	toe	AA5083_8	180	175,191,75	2.2553	5.2435
Al/steel_9	103	337,235	2.0128	5.5279	root	AA5083_9	180	286,420.25	2.2553	5.457
Al/steel_10	103	224,644	2.0128	5.3515	root	AA5083_10	180	27,484.75	2.2553	5.4391
Al/steel_11	103	262,865	2.0128	5.4197	toe	AA5083_11	180	318,084.75	2.2553	5.5025
Al/steel_12	103	508,069	2.0128	5.7059	root	AA5083_12	180	337,226.5	2.2553	5.5279
Al/steel_13	103	418,474	2.0128	5.6217	root	AA5083_13	180	342,419	2.2553	5.5346
Al/steel_14	103	264,370	2.0128	5.4222	toe	AA5083_14	245	78,532.75	2.3892	4.8951
Al/steel_15	103	141,606	2.0128	5.1511	toe	AA5083_15	245	78,859.00	2.3892	4.8969
Al/steel_16	103	170,746	2.0128	5.2324	root	AA5083_16	245	83,264.00	2.3892	4.9205
Al/steel_17	135	21,845	2.1303	4.3394	root	AA5083_17	245	66,117.75	2.3892	4.8203
Al/steel_18	135	221,116	2.1303	5.3446	root	AA5083_18	245	61,106.75	2.3892	4.7861
Al/steel_19	135	136,318	2.1303	5.1346	toe	AA5083_19	245	79,962.5	2.3892	4.9029
Al/steel_20	135	56,302	2.1303	4.7505	toe	AA5083_20	245	64,683	2.3892	4.8108
Al/steel_21	135	58,127	2.1303	4.7644	root	AA5083_21	330	22,573.75	2.5185	4.3536
Al/steel_22	135	40,012	2.1303	4.6022	root	AA5083_22	330	14,957.75	2.5185	4.1749
Al/steel_23	135	80,025	2.1303	4.9032	toe	AA5083_23	330	14,274.75	2.5185	4.1546
Al/steel_24	135	65,245	2.1303	4.8145	root	AA5083_24	330	19,495.75	2.5185	4.2899
Al/steel_25	175	16,508	2.243	4.2177	root	AA5083_25	330	17,583.50	2.5185	4.2451
Al/steel_26	175	6647	2.243	3.8226	root	AA5083_26	330	19,124.75	2.5185	4.2816
Al/steel_27	175	12,049	2.243	4.081	root					
Al/steel_28	175	9300	2.243	3.9685	root					
Al/steel_29	175	17,890	2.243	4.2526	toe					
Al/steel_30	175	27,472	2.243	4.4389	root					
Al/steel_31	175	12,011	2.243	4.0796	root					
Al/steel_32	175	15,615	2.243	4.1935	root					

**Table 6:** Values determined on the basis of experimental studies slope and constant describing the S–N fatigue diagrams for BM and transition joint Al/steel.

	M	A	R^2^	σ_max_ for N = 5 × 10^6^
	-	-	-	MPa
AA5083 (BM)	4.65	15.95	0.98	99.5
Al/Fe Transition Joint	5.18	15.80	0.87	63.1

## Data Availability

The raw/processed data required to reproduce these findings cannot be shared at this time due to technical or time limitations.
